# Supplementary material for: Structures of co-transcriptional RNA capping enzymes on paused transcription complex
Source: Nat Commun. 2024 May 30;15:4622. doi: 10.1038/s41467-024-48963-1 (PMC11139899; doi:10.1038/s41467-024-48963-1)
Supplement: Supplementary file 1 — Supplementary Information [file 41467_2024_48963_MOESM1_ESM.pdf]

Supplementary Information For

Structures of co-transcriptional RNA capping enzymes on paused transcription complex

Yan Li[1*], Qianmin Wang[1*], Yanhui Xu[1,2], and Ze Li[1,2†]

[1]Fudan University Shanghai Cancer Center, Institutes of Biomedical Sciences, State Key Laboratory of Genetic Engineering and Shanghai Key Laboratory of Medical Epigenetics, Shanghai Medical College of Fudan University, Shanghai 200032, China.

[2]The International Co-laboratory of Medical Epigenetics and Metabolism, Ministry of Science and Technology, China, Department of Systems Biology for Medicine, School of Basic Medical Sciences, Shanghai Medical College of Fudan University, Shanghai 200032, China.

* The authors contributed to the study equally

† To whom correspondence should be addressed. E-mail: zeli@fudan.edu.cn

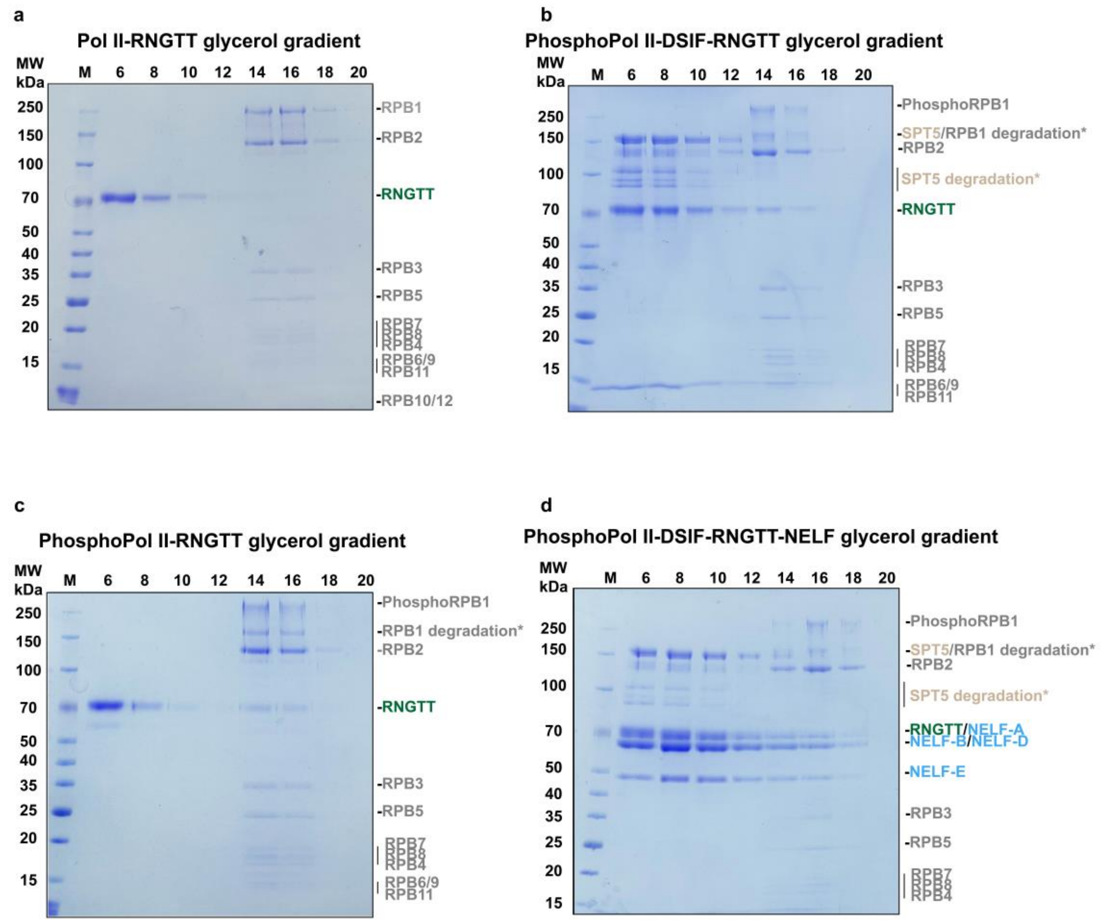

Supplementary Fig. 1 Pol II-RNGTT Complex assembly through glycerol density gradient ultracentrifugation. a-d Indicated fractions of the assembly of Pol II-RNGTT complexes containing different components through glycerol density gradient ultracentrifugation.

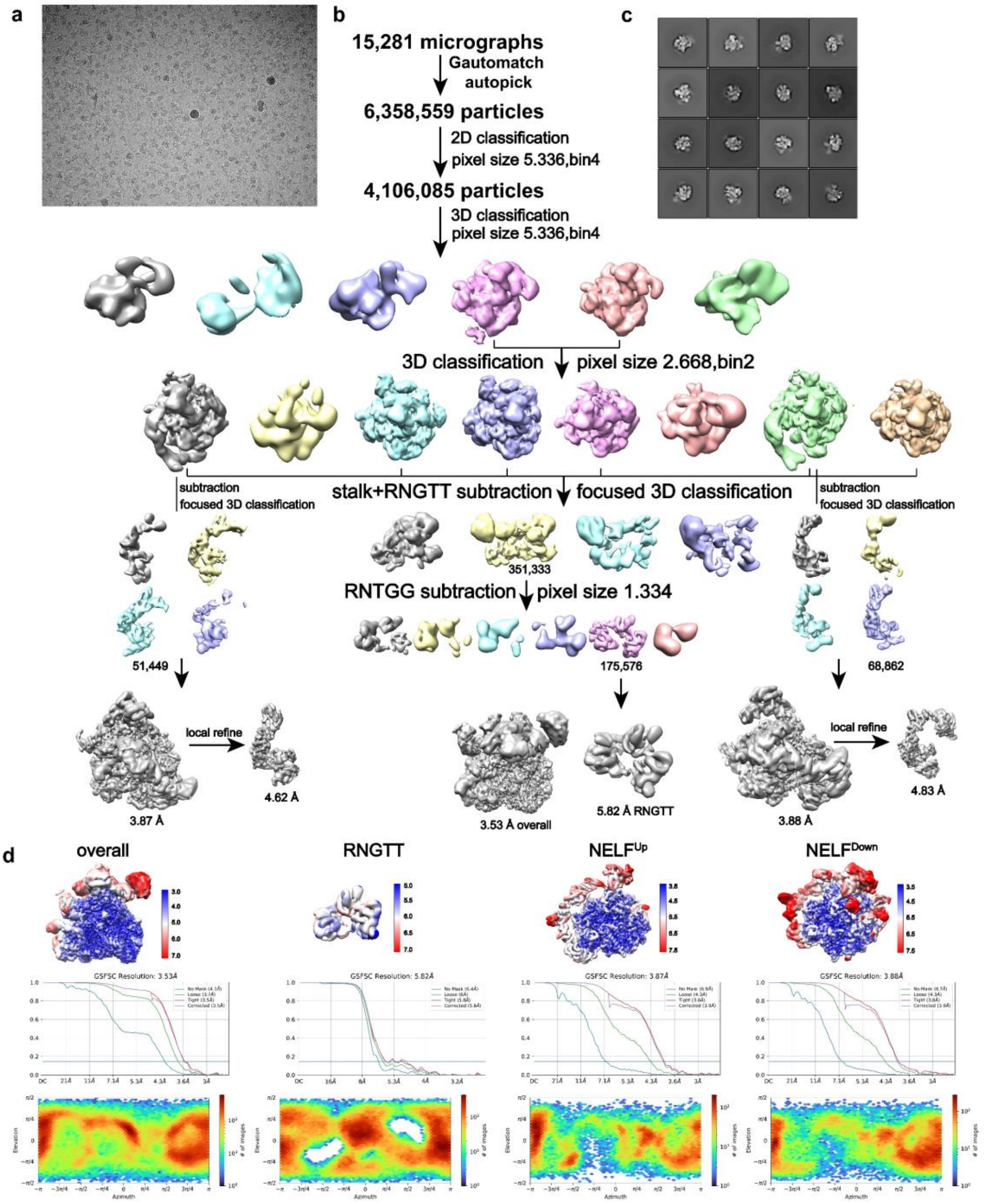

Supplementary Fig. 2 Data collection and image processing of PEC-RNGTT complex. a Representative cryo-EM micrograph. **b** Flow-charts of the cryo-EM image processing and 3D reconstructions of the human PEC-RNGTT. **c** 2D classification from at least three times repeatedly of the human PEC-bound RNGTT. **d** Local resolution estimation, FSC curves and orientation of the cryo-EM reconstructions of Pol II-DSIF-RNGTT complex, masked RNGTT, PEC-RNGTT complex with NELF^{Up} conformation and PEC-RNGTT complex with NELF^{Down} conformation.

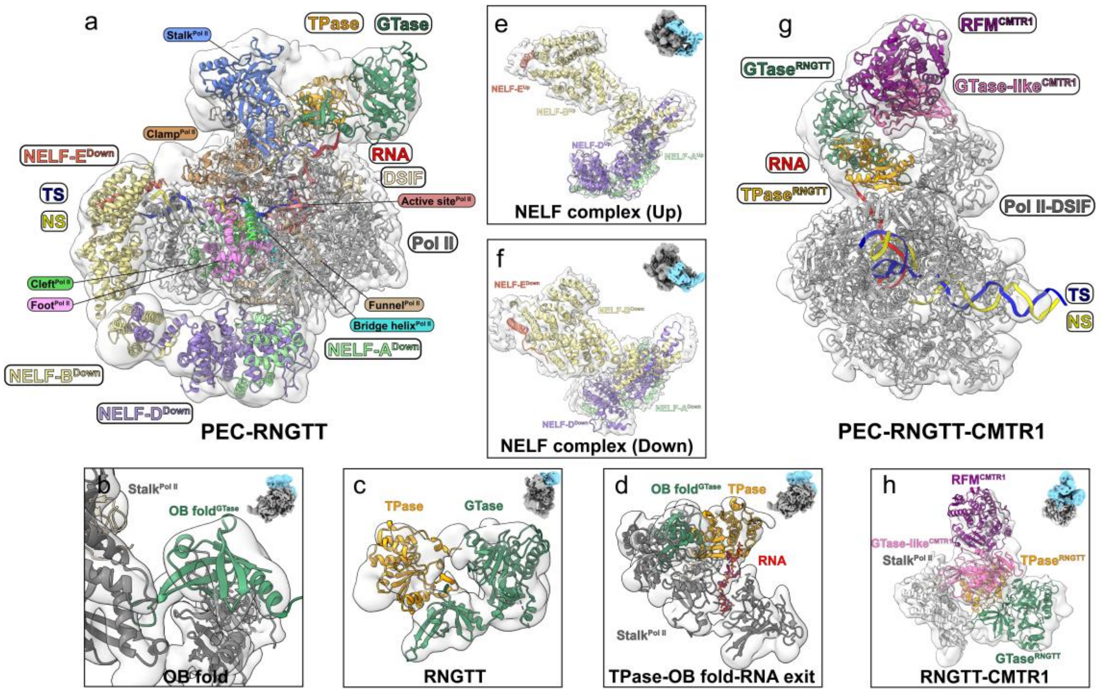

Supplementary Fig. 3 Cryo-EM maps and structural models of PEC-RNGTT and PEC-RNGTT-CMTR1 complex. a, g Overall cryo-EM maps of PEC-RNGTT(**a**) and PEC-RNGTT-CMTR1 complex(**g**) fitted with individual model. Cryo-EM maps are shown in transparent surfaces with models shown in cartoon. Important components of Pol II are colored and labeled around in corresponding colors. **b-f, h** Focused refinement cryo-EM maps in different regions are shown in boxed panels. The maps at the top right indicate the masked regions.

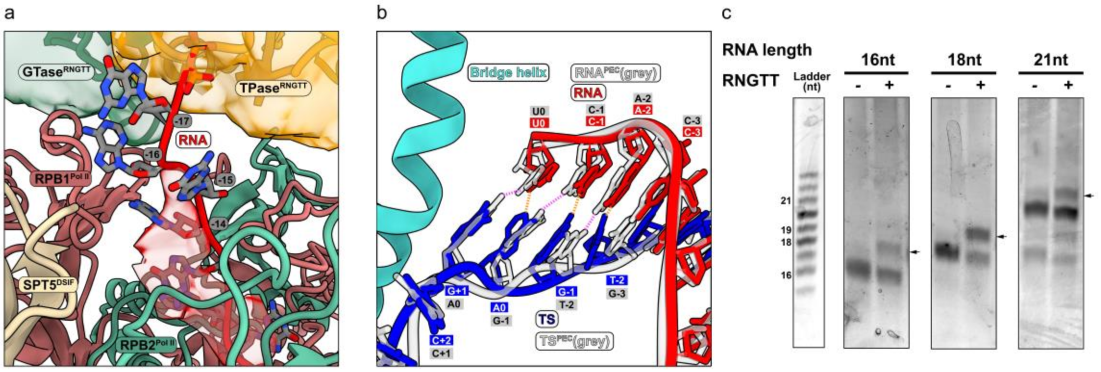

Supplementary Fig. 4 Detailed conformation of RNA in PEC-RNGTT complex. **a** RNA at the exit tunnel. Cryo-EM density of RNA is shown in red semi-transparently with its model fitted in. About 15 nt RNA could be tracked in the density. Other components are showed as ribbons with labels in corresponding colors. **b** Structural comparison of RNA at the active site with PEC. Nucleic acids of PEC-RNGTT are colored and nucleic acids of PEC are semi-transparent grey. Base paring between RNA and TS of PEC-RNGTT is displayed as dashes in orange, and base paring of PEC is displayed as dashes in magenta. **c** *In vitro* guanylylation assay in the context of phosphorylated Pol II-DSIF-RNGTT complex for 16 nt, 18 nt and 21 nt RNAs. Corresponding ladder is on the left. Bands of guanylylated RNAs are pointed out with arrows. The experiment was repeated at least three times. Source data are provided as a Source Data file.

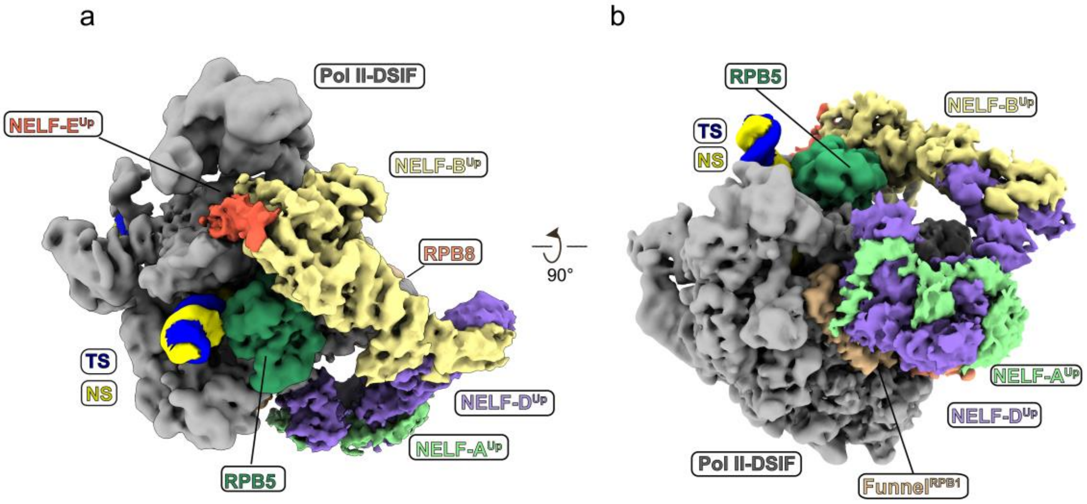

Supplementary Fig. 5 Overall structure of NELF in the "Up" state conformation. a Overall structure of NELF in "Up" state conformation in PEC-RNGTT in the same view with Fig.3a. **b** Overall structure of NELF in "Up" state conformation in PEC-RNGTT in the same view with Fig. 3a.

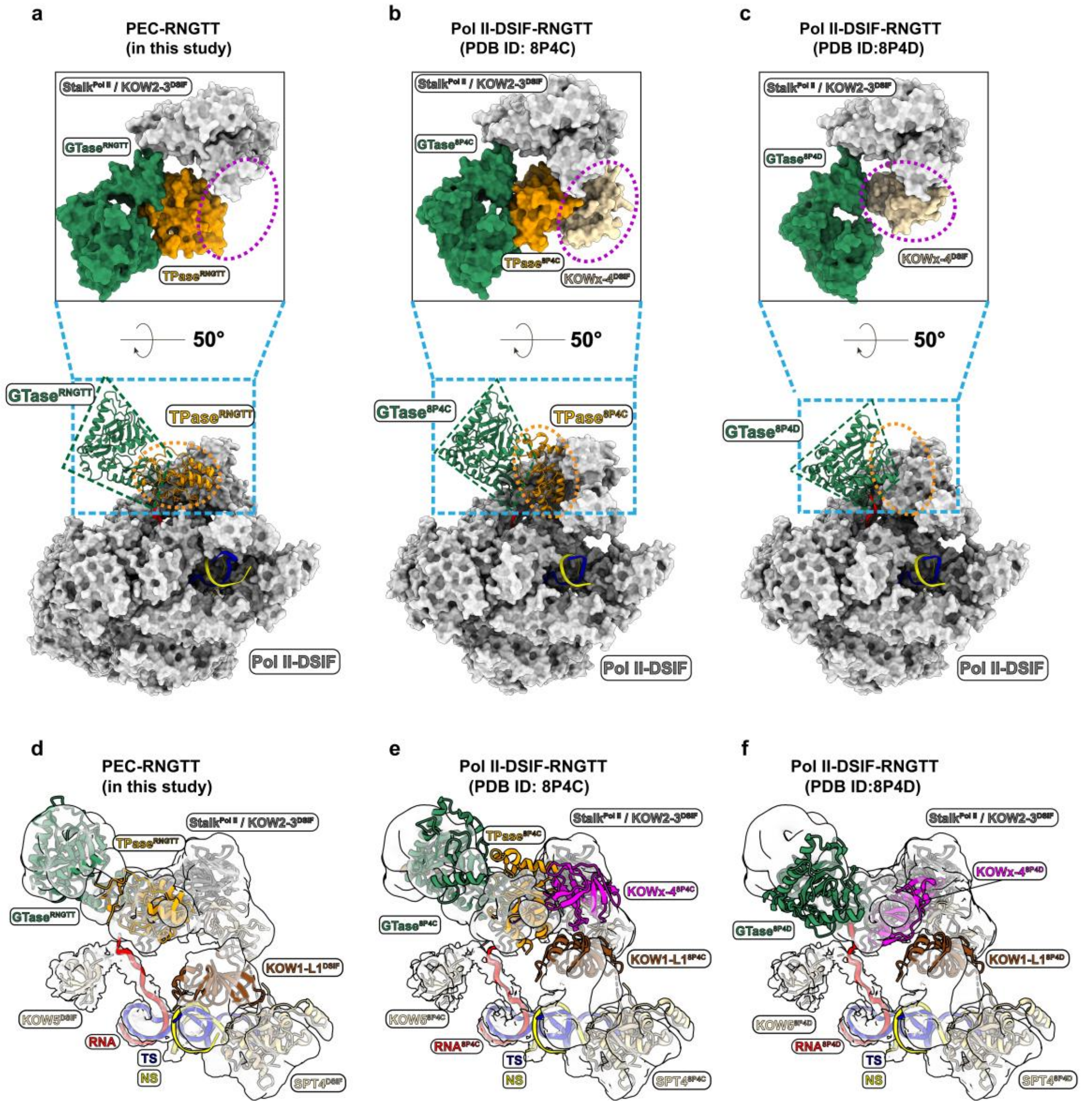

Supplementary Fig. 6 Structural comparison of PEC-RNGTT with two transcribing Pol II-DSIF-RNGTT complexes. a-c Location of $\text{TPase}^{\text{RNGTT}}$, $\text{GTase}^{\text{RNGTT}}$ and $\text{KOWx-KOW4}^{\text{DSIF}}$ of the three structures in two views. **a** PEC-RNGTT; **b** Transcribing Pol II-DSIF- RNGTT complex (PDB ID: 8P4C); **c** Transcribing Pol II-DSIF-RNGTT complex without visible $\text{TPase}^{\text{RNGTT}}$(PDB ID: 8P4D). **d-f** Models of PEC-RNGTT and two transcribing Pol II-DSIF-RNGTT complexes fit into cryo-EM map of PEC-RNGTT. **d** PEC-RNGTT; **e** Transcribing Pol II-DSIF-RNGTT complex (PDB ID: 8P4C); **f** Transcribing Pol II-DSIF-RNGTT complex without visible $\text{TPase}^{\text{RNGTT}}$ (PDB ID: 8P4D).

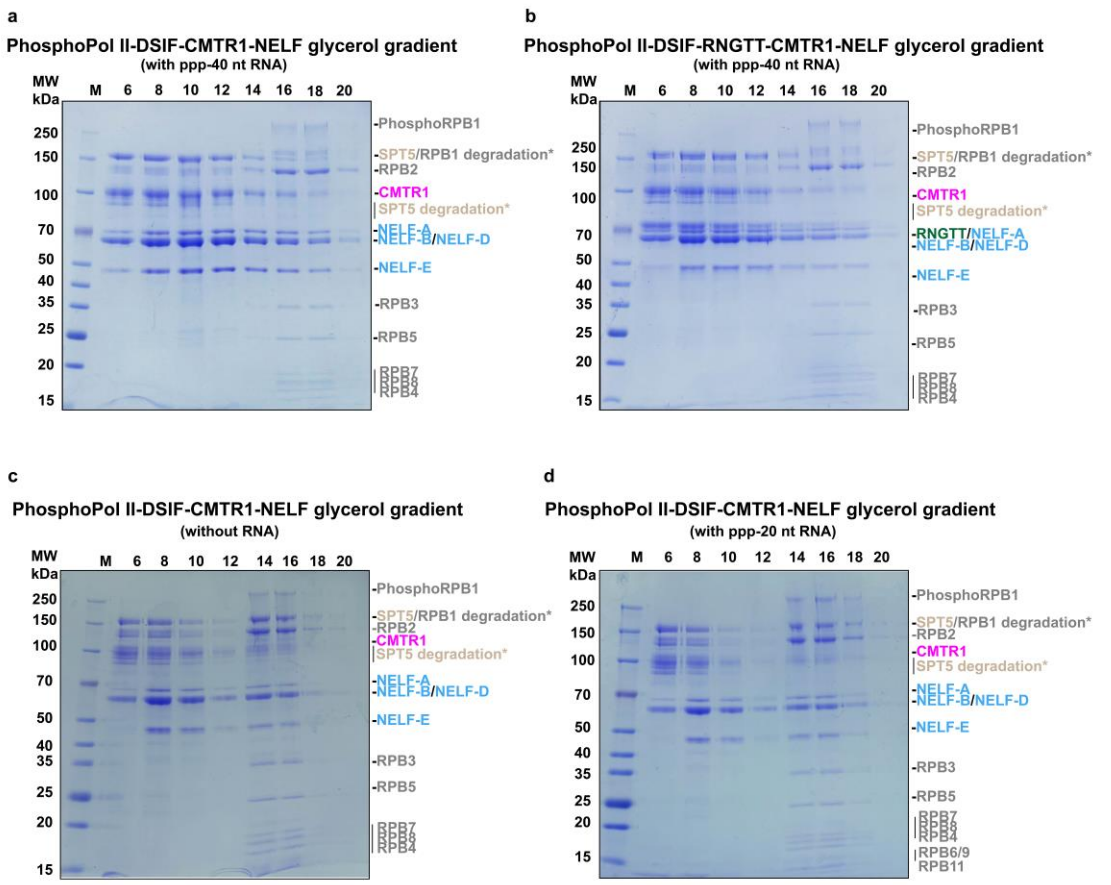

Supplementary Fig. 7 Pol II-CMTR1 Complex assembly through glycerol density gradient ultracentrifugation. a-d Indicated fractions of the assembly of Pol II-CMTR1 complexes containing different components through glycerol density gradient ultracentrifugation.

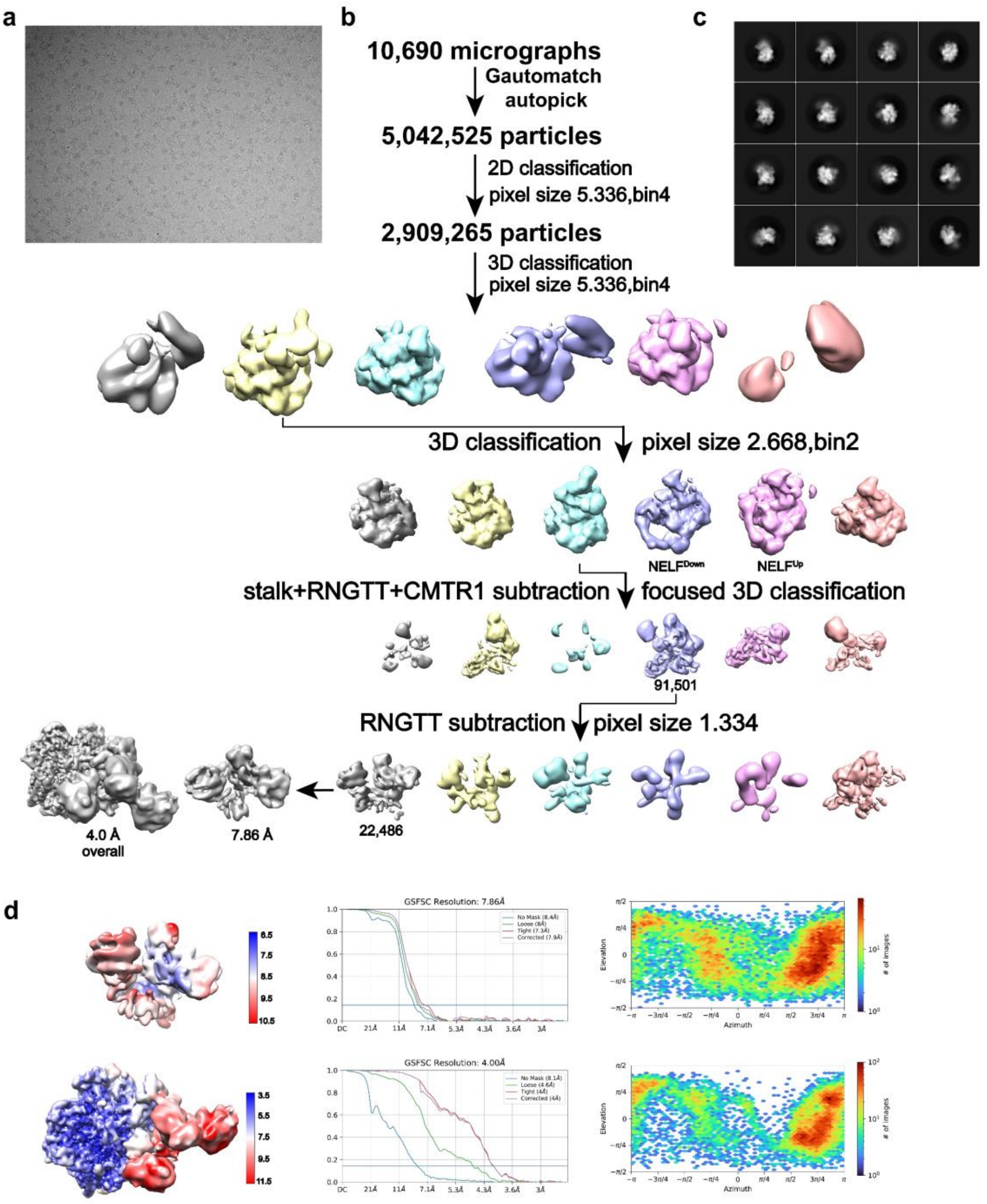

Supplementary Fig. 8 Data collection and image processing of PEC-RNGTT-CMTR1 complex. a Representative cryo-EM images. **b** Flow-charts of the cryo-EM image processing and 3D reconstructions of the human PEC-RNGTT-CMTR1 complex. **c** 2D classification from at least three times repeatedly of the human PEC-RNGTT-CMTR1 complex. **d** Local resolution estimation, FSC curves and orientations of the cryo-EM reconstructions of PEC-RNGTT-CMTR1 and focused refinement RNGTT-CMTR1.

Supplementary Table 1. Statistics of cryo-EM data collection and refinement.

	PEC-RNGTT (NELF^{Down})		PEC-RNGTT-CMTR1	
	**Overall** (EMDB-37352) (PDB-8W8E)	**RNGTT**	**Overall** (EMDB-37353) (PDB-8W8F)	**RNGTT-CMTR1**
**Data collection and processing**				
Magnification	64,000 x	64,000 x	64,000 x	64,000 x
Voltage (kV)	300	300	300	300
Electron exposure (e^{-}/Å^2)	50	50	50	50
Defocus range (μm)	-1.5 ~ -2.5	-1.5 ~ -2.5	-1.5 ~ -2.5	-1.5 ~ -2.5
Pixel size (Å)	1.334	1.334	1.334	1.334
Symmetry imposed	C1	C1	C1	C1
Initial particle images (no.)	6,358,559	6,358,559	5,042,525	5,042,525
Final particle images (no.)	68,862	175,576	22,486	22,486
Map resolution (Å)	3.9	5.8	4.0	7.9
FSC threshold	0.143	0.143	0.143	0.143
Map resolution range (Å)	3.0~7.0	5.0~7.0	6.5~11.5	3.5~11.5
**Refinement**				
Model resolution (Å)	3.9		4.1	
FSC threshold	0.143		0.143	
Model composition				
Non-hydrogen atoms	50,901		46,667	
Protein residues	6,183		5,587	
Nucleotides	95		95	
Ligands	GTP: 1<br>Zn: 8<br>Mg: 1		GTP: 1<br>Zn: 8<br>Mg: 1	
B factors (Å^2)				
Protein	41.25		36.68	
Nucleotide	68.00		68.00	
Ligand	30.91		30.47	
R.m.s deviations				
Bond lengths (Å)	0.003		0.002	
Bond angles (°)	0.590		0.539	
Validation				
MolProbity score	2.32		2.22	
Clash score	9.57		8.34	
Poor rotamers (%)	3.56		3.62	
Ramachandran plot				
Favored (%)	94.03		95.11	
Allowed (%)	5.81		4.80	
Disallowed (%)	0.16		0.09	

Supplementary Table 2. ***In vitro*** **transcribed RNA sequence used in guanylylation assay.**

RNA length	Sequence
15 nt	5′-GGGAGAGAACCCACU-3′
16 nt	5′-GGGAGAGGAACCCACU-3′
17 nt	5′-GGGAGAGGGAACCCACU-3′
18 nt	5′-GGGAAGAGGGAACCCACU-3′
19 nt	5′-GGGAAGAAGGGAACCCACU-3′
20 nt	5′-GGGAAGAGAGGGAACCCACU-3′
21 nt	5′-GGGAAGGAGAGGGAACCCACU-3′
22 nt	5′-GGGAACGGAGAGGGAACCCACU-3′
23 nt	5′-GGGAACCGGAGAGGGAACCCACU-3′
26 nt	5′-GGGAAUAACCGGAGAGGGAACCCACU-3′
28 nt	5′-GGGCUAAUAACCGGAGAGGGAACCCACU-3′
33 nt	5′-GGGUGCGUCUAAUAACCGGAGAGGGAACCCACU-3′
